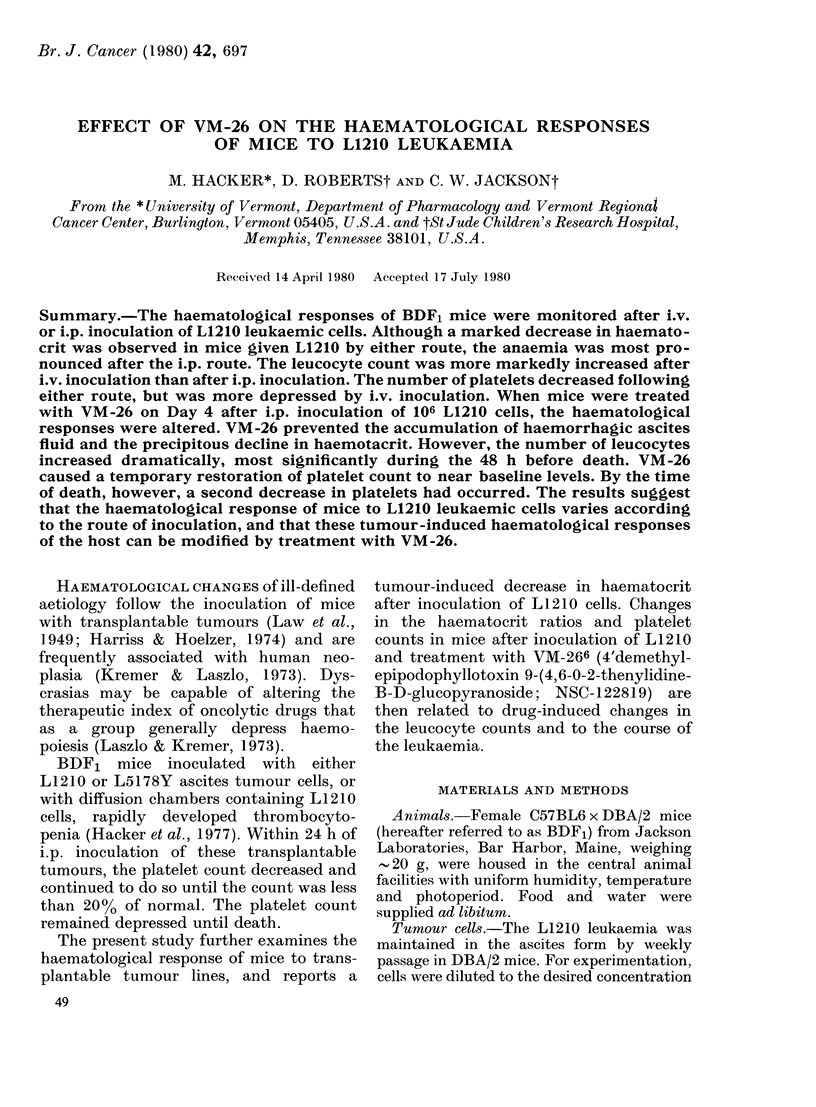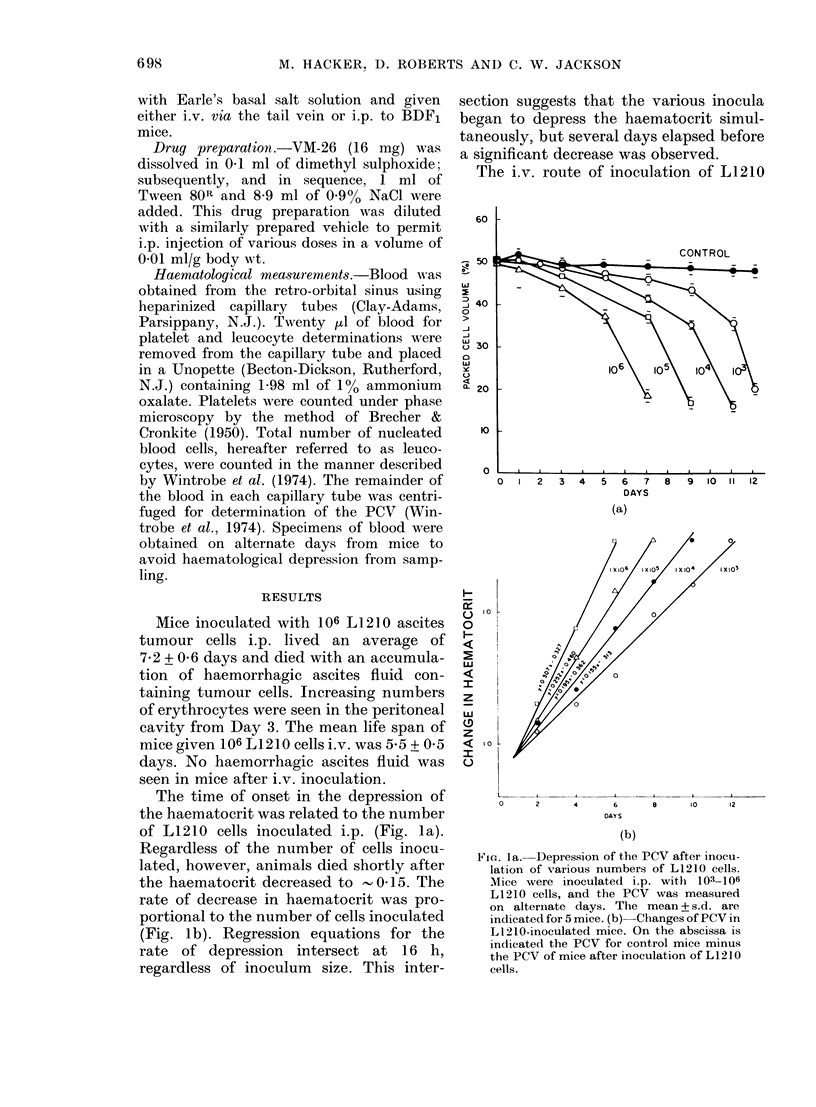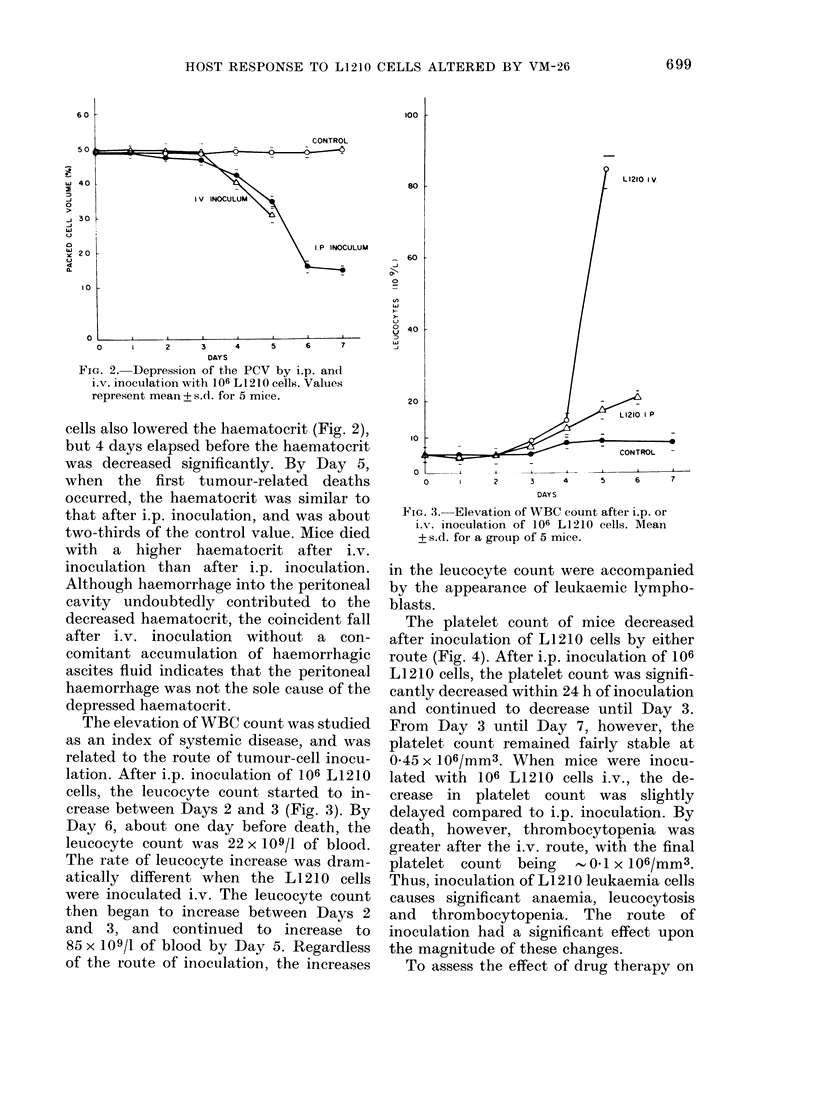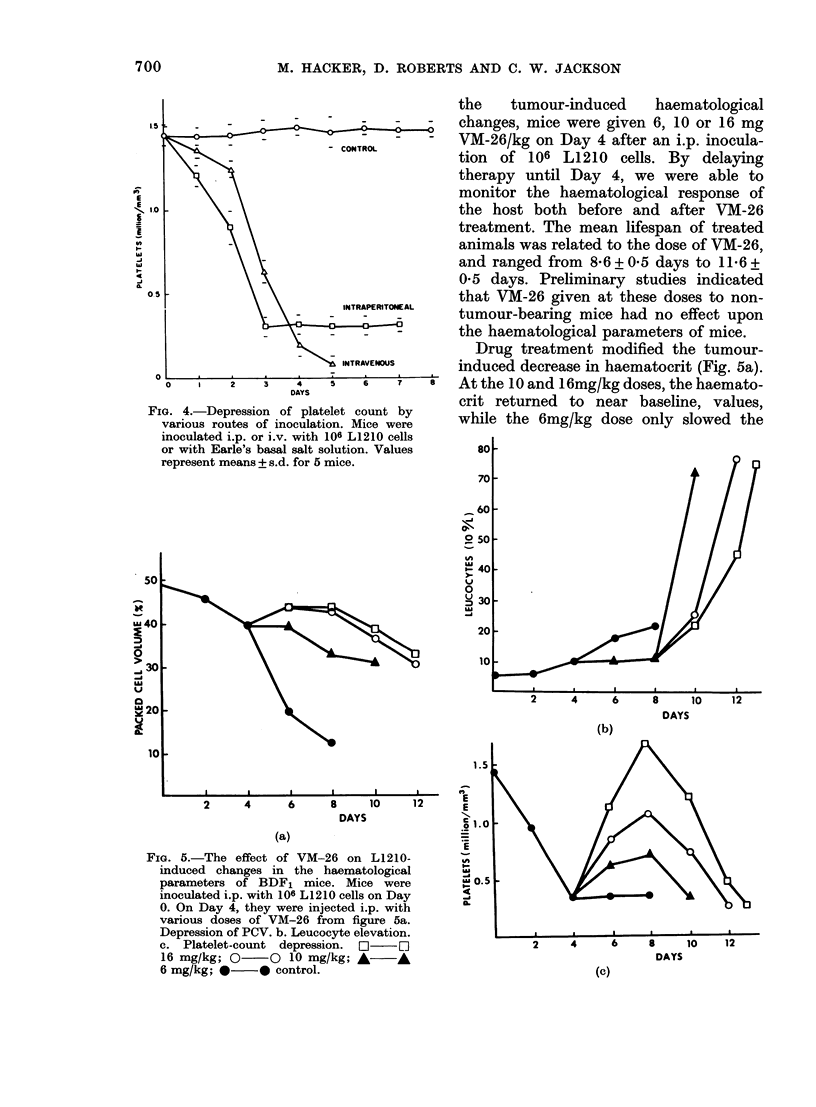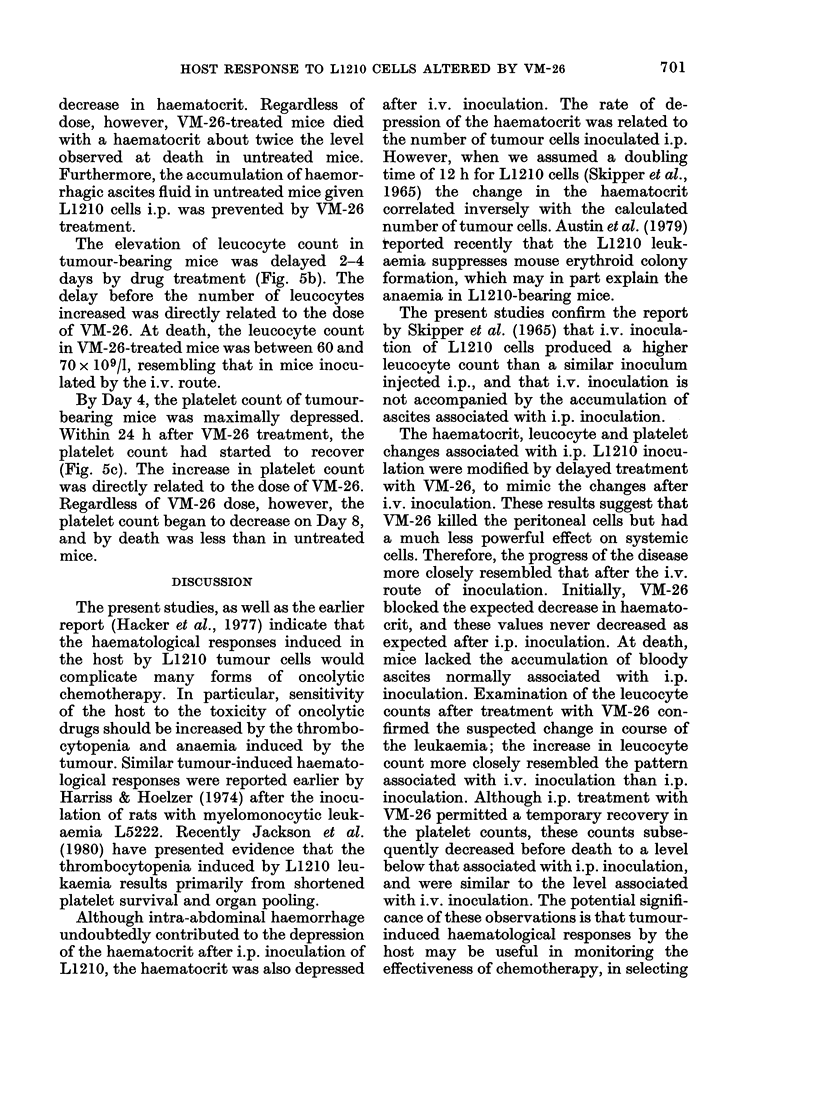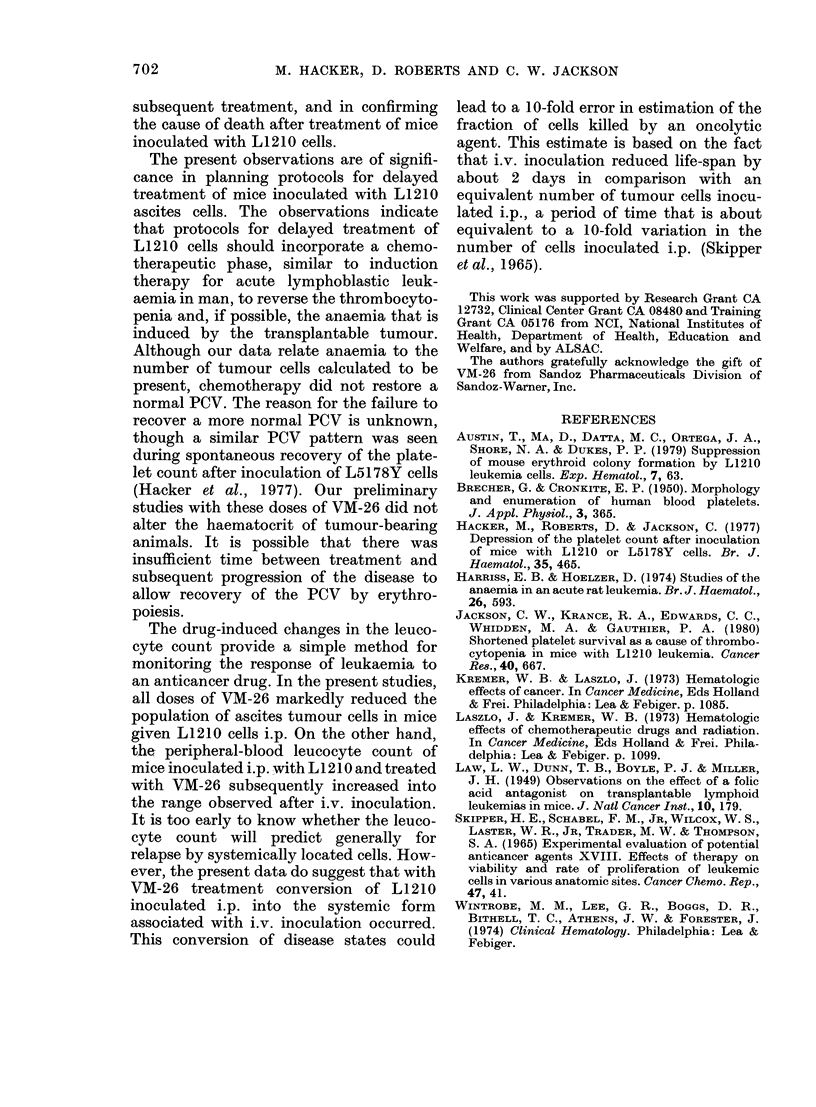# Effect of VM-26 on the haematological responses of mice to L1210 leukaemia.

**DOI:** 10.1038/bjc.1980.304

**Published:** 1980-11

**Authors:** M. Hacker, D. Roberts, C. W. Jackson

## Abstract

The haematological responses of BDF1 mice were monitored after i.v. or i.p. inoculation of L1210 leukaemic cells. Although a marked decrease in haematocrit was observed in mice given L1210 by either route, the anaemia was most pronounced after the i.p. route. The leucocyte count was more markedly increased after i.v. inoculation than after i.p. inoculation. The number of platelets decreased following either route, but was more depressed by i.v. inoculation. When mice were treated with VM-26 on Day 4 and i.p. inoculation of 10(6) L1210 cells, the haematological responses were altered. VM-26 prevented the accumulation of haemorrhagic ascites fluid and the precipitous decline in haemotacrit. However, the number of leucocytes increased dramatically, most significantly during the 48 h before death. VM-26 caused a temporary restoration of platelet count to near baseline levels. By the time of death, however, a second decrease in platelets had occurred. The results suggest that the haematological response of mice to L1210 leukaemic cells varies according to the route of inoculation, and that these tumour-induced haematological responses of the host can be modified by treatment with VM-26.


*Br. J. Cancer* (1980) **42**, 697

### EFFECT OF VM-26 ON THE HAEMATOLOGICAL RESPONSES OF MICE TO L1210 LEUKAEMIA

M. HACKER*, D. ROBERTS† AND C. W. JACKSON†

*From the *University of Vermont, Department of Pharmacology and Vermont Regional Cancer Center, Burlington, Vermont* 05405, *U.S.A. and †St Jude Children's Research Hospital, Memphis, Tennessee* 38101, *U.S.A.*

Received 14 April 1980 Accepted 17 July 1980

**Summary.—The haematological responses of $BDF_1$ mice were monitored after i.v. or i.p. inoculation of L1210 leukaemic cells. Although a marked decrease in haematocrit was observed in mice given L1210 by either route, the anaemia was most pronounced after the i.p. route. The leucocyte count was more markedly increased after i.v. inoculation than after i.p. inoculation. The number of platelets decreased following either route, but was more depressed by i.v. inoculation. When mice were treated with VM-26 on Day 4 after i.p. inoculation of $10^6$ L1210 cells, the haematological responses were altered. VM-26 prevented the accumulation of haemorrhagic ascites fluid and the precipitous decline in haemotacrit. However, the number of leucocytes increased dramatically, most significantly during the 48 h before death. VM-26 caused a temporary restoration of platelet count to near baseline levels. By the time of death, however, a second decrease in platelets had occurred. The results suggest that the haematological response of mice to L1210 leukaemic cells varies according to the route of inoculation, and that these tumour-induced haematological responses of the host can be modified by treatment with VM-26.**

HAEMATOLOGICAL CHANGES of ill-defined aetiology follow the inoculation of mice with transplantable tumours (Law *et al.*, 1949; Harriss & Hoelzer, 1974) and are frequently associated with human neoplasia (Kremer & Laszlo, 1973). Dyscrasias may be capable of altering the therapeutic index of oncolytic drugs that as a group generally depress haemopoiesis (Laszlo & Kremer, 1973).

$BDF_1$ mice inoculated with either L1210 or L5178Y ascites tumour cells, or with diffusion chambers containing L1210 cells, rapidly developed thrombocytopenia (Hacker *et al.*, 1977). Within 24 h of i.p. inoculation of these transplantable tumours, the platelet count decreased and continued to do so until the count was less than 20% of normal. The platelet count remained depressed until death.

The present study further examines the haematological response of mice to transplantable tumour lines, and reports a tumour-induced decrease in haematocrit after inoculation of L1210 cells. Changes in the haematocrit ratios and platelet counts in mice after inoculation of L1210 and treatment with VM-26[6] (4'demethyl-epipodophyllotoxin 9-(4,6-0-2-thenylidine-B-D-glucopyranoside; NSC-122819) are then related to drug-induced changes in the leucocyte counts and to the course of the leukaemia.

### MATERIALS AND METHODS

*Animals.*—Female C57BL6 × DBA/2 mice (hereafter referred to as $BDF_1$) from Jackson Laboratories, Bar Harbor, Maine, weighing ~20 g, were housed in the central animal facilities with uniform humidity, temperature and photoperiod. Food and water were supplied *ad libitum.*

*Tumour cells.*—The L1210 leukaemia was maintained in the ascites form by weekly passage in DBA/2 mice. For experimentation, cells were diluted to the desired concentration with Earle's basal salt solution and given either i.v. *via* the tail vein or i.p. to $BDF_1$ mice.

*Drug preparation.*—VM-26 (16 mg) was dissolved in 0·1 ml of dimethyl sulphoxide; subsequently, and in sequence, 1 ml of Tween $80^R$ and 8·9 ml of 0·9% NaCl were added. This drug preparation was diluted with a similarly prepared vehicle to permit i.p. injection of various doses in a volume of 0·01 ml/g body wt.

*Haematological measurements.*—Blood was obtained from the retro-orbital sinus using heparinized capillary tubes (Clay-Adams, Parsippany, N.J.). Twenty $\mu$l of blood for platelet and leucocyte determinations were removed from the capillary tube and placed in a Unopette (Becton-Dickson, Rutherford, N.J.) containing 1·98 ml of 1% ammonium oxalate. Platelets were counted under phase microscopy by the method of Brecher & Cronkite (1950). Total number of nucleated blood cells, hereafter referred to as leucocytes, were counted in the manner described by Wintrobe *et al.* (1974). The remainder of the blood in each capillary tube was centrifuged for determination of the PCV (Wintrobe *et al.*, 1974). Specimens of blood were obtained on alternate days from mice to avoid haematological depression from sampling.

### RESULTS

Mice inoculated with $10^6$ L1210 ascites tumour cells i.p. lived an average of $7{\cdot}2 \pm 0{\cdot}6$ days and died with an accumulation of haemorrhagic ascites fluid containing tumour cells. Increasing numbers of erythrocytes were seen in the peritoneal cavity from Day 3. The mean life span of mice given $10^6$ L1210 cells i.v. was $5{\cdot}5 \pm 0{\cdot}5$ days. No haemorrhagic ascites fluid was seen in mice after i.v. inoculation.

The time of onset in the depression of the haematocrit was related to the number of L1210 cells inoculated i.p. (Fig. 1a). Regardless of the number of cells inoculated, however, animals died shortly after the haematocrit decreased to ~0·15. The rate of decrease in haematocrit was proportional to the number of cells inoculated (Fig. 1b). Regression equations for the rate of depression intersect at 16 h, regardless of inoculum size. This intersection suggests that the various inocula began to depress the haematocrit simultaneously, but several days elapsed before a significant decrease was observed.

The i.v. route of inoculation of L1210

FIG. 1a.—Depression of the PCV after inoculation of various numbers of L1210 cells. Mice were inoculated i.p. with $10^3$–$10^6$ L1210 cells, and the PCV was measured on alternate days. The mean ± s.d. are indicated for 5 mice. (b)—Changes of PCV in L1210-inoculated mice. On the abscissa is indicated the PCV for control mice minus the PCV of mice after inoculation of L1210 cells.

FIG. 2.—Depression of the PCV by i.p. and i.v. inoculation with $10^6$ L1210 cells. Values represent mean ± s.d. for 5 mice.

cells also lowered the haematocrit (Fig. 2), but 4 days elapsed before the haematocrit was decreased significantly. By Day 5, when the first tumour-related deaths occurred, the haematocrit was similar to that after i.p. inoculation, and was about two-thirds of the control value. Mice died with a higher haematocrit after i.v. inoculation than after i.p. inoculation. Although haemorrhage into the peritoneal cavity undoubtedly contributed to the decreased haematocrit, the coincident fall after i.v. inoculation without a concomitant accumulation of haemorrhagic ascites fluid indicates that the peritoneal haemorrhage was not the sole cause of the depressed haematocrit.

The elevation of WBC count was studied as an index of systemic disease, and was related to the route of tumour-cell inoculation. After i.p. inoculation of $10^6$ L1210 cells, the leucocyte count started to increase between Days 2 and 3 (Fig. 3). By Day 6, about one day before death, the leucocyte count was $22 \times 10^9$/l of blood. The rate of leucocyte increase was dramatically different when the L1210 cells were inoculated i.v. The leucocyte count then began to increase between Days 2 and 3, and continued to increase to $85 \times 10^9$/l of blood by Day 5. Regardless of the route of inoculation, the increases

FIG. 3.—Elevation of WBC count after i.p. or i.v. inoculation of $10^6$ L1210 cells. Mean ± s.d. for a group of 5 mice.

in the leucocyte count were accompanied by the appearance of leukaemic lymphoblasts.

The platelet count of mice decreased after inoculation of L1210 cells by either route (Fig. 4). After i.p. inoculation of $10^6$ L1210 cells, the platelet count was significantly decreased within 24 h of inoculation and continued to decrease until Day 3. From Day 3 until Day 7, however, the platelet count remained fairly stable at $0{\cdot}45 \times 10^6/mm^3$. When mice were inoculated with $10^6$ L1210 cells i.v., the decrease in platelet count was slightly delayed compared to i.p. inoculation. By death, however, thrombocytopenia was greater after the i.v. route, with the final platelet count being $\sim 0{\cdot}1 \times 10^6/mm^3$. Thus, inoculation of L1210 leukaemia cells causes significant anaemia, leucocytosis and thrombocytopenia. The route of inoculation had a significant effect upon the magnitude of these changes.

To assess the effect of drug therapy on

FIG. 4.—Depression of platelet count by various routes of inoculation. Mice were inoculated i.p. or i.v. with $10^6$ L1210 cells or with Earle's basal salt solution. Values represent means ± s.d. for 5 mice.

the tumour-induced haematological changes, mice were given 6, 10 or 16 mg VM-26/kg on Day 4 after an i.p. inoculation of $10^6$ L1210 cells. By delaying therapy until Day 4, we were able to monitor the haematological response of the host both before and after VM-26 treatment. The mean lifespan of treated animals was related to the dose of VM-26, and ranged from 8·6 ± 0·5 days to 11·6 ± 0·5 days. Preliminary studies indicated that VM-26 given at these doses to non-tumour-bearing mice had no effect upon the haematological parameters of mice.

Drug treatment modified the tumour-induced decrease in haematocrit (Fig. 5a). At the 10 and 16mg/kg doses, the haematocrit returned to near baseline, values, while the 6mg/kg dose only slowed the

FIG. 5.—The effect of VM-26 on L1210-induced changes in the haematological parameters of $BDF_1$ mice. Mice were inoculated i.p. with $10^6$ L1210 cells on Day 0. On Day 4, they were injected i.p. with various doses of VM-26 from figure 5a. Depression of PCV. b. Leucocyte elevation. c. Platelet-count depression. □——□ 16 mg/kg; ○——○ 10 mg/kg; ▲——▲ 6 mg/kg; ●——● control.

decrease in haematocrit. Regardless of dose, however, VM-26-treated mice died with a haematocrit about twice the level observed at death in untreated mice. Furthermore, the accumulation of haemorrhagic ascites fluid in untreated mice given L1210 cells i.p. was prevented by VM-26 treatment.

The elevation of leucocyte count in tumour-bearing mice was delayed 2–4 days by drug treatment (Fig. 5b). The delay before the number of leucocytes increased was directly related to the dose of VM-26. At death, the leucocyte count in VM-26-treated mice was between 60 and $70 \times 10^9$/l, resembling that in mice inoculated by the i.v. route.

By Day 4, the platelet count of tumour-bearing mice was maximally depressed. Within 24 h after VM-26 treatment, the platelet count had started to recover (Fig. 5c). The increase in platelet count was directly related to the dose of VM-26. Regardless of VM-26 dose, however, the platelet count began to decrease on Day 8, and by death was less than in untreated mice.

### DISCUSSION

The present studies, as well as the earlier report (Hacker *et al.*, 1977) indicate that the haematological responses induced in the host by L1210 tumour cells would complicate many forms of oncolytic chemotherapy. In particular, sensitivity of the host to the toxicity of oncolytic drugs should be increased by the thrombocytopenia and anaemia induced by the tumour. Similar tumour-induced haematological responses were reported earlier by Harriss & Hoelzer (1974) after the inoculation of rats with myelomonocytic leukaemia L5222. Recently Jackson *et al.* (1980) have presented evidence that the thrombocytopenia induced by L1210 leukaemia results primarily from shortened platelet survival and organ pooling.

Although intra-abdominal haemorrhage undoubtedly contributed to the depression of the haematocrit after i.p. inoculation of L1210, the haematocrit was also depressed after i.v. inoculation. The rate of depression of the haematocrit was related to the number of tumour cells inoculated i.p. However, when we assumed a doubling time of 12 h for L1210 cells (Skipper *et al.*, 1965) the change in the haematocrit correlated inversely with the calculated number of tumour cells. Austin *et al.* (1979) reported recently that the L1210 leukaemia suppresses mouse erythroid colony formation, which may in part explain the anaemia in L1210-bearing mice.

The present studies confirm the report by Skipper *et al.* (1965) that i.v. inoculation of L1210 cells produced a higher leucocyte count than a similar inoculum injected i.p., and that i.v. inoculation is not accompanied by the accumulation of ascites associated with i.p. inoculation.

The haematocrit, leucocyte and platelet changes associated with i.p. L1210 inoculation were modified by delayed treatment with VM-26, to mimic the changes after i.v. inoculation. These results suggest that VM-26 killed the peritoneal cells but had a much less powerful effect on systemic cells. Therefore, the progress of the disease more closely resembled that after the i.v. route of inoculation. Initially, VM-26 blocked the expected decrease in haematocrit, and these values never decreased as expected after i.p. inoculation. At death, mice lacked the accumulation of bloody ascites normally associated with i.p. inoculation. Examination of the leucocyte counts after treatment with VM-26 confirmed the suspected change in course of the leukaemia; the increase in leucocyte count more closely resembled the pattern associated with i.v. inoculation than i.p. inoculation. Although i.p. treatment with VM-26 permitted a temporary recovery in the platelet counts, these counts subsequently decreased before death to a level below that associated with i.p. inoculation, and were similar to the level associated with i.v. inoculation. The potential significance of these observations is that tumour-induced haematological responses by the host may be useful in monitoring the effectiveness of chemotherapy, in selecting subsequent treatment, and in confirming the cause of death after treatment of mice inoculated with L1210 cells.

The present observations are of significance in planning protocols for delayed treatment of mice inoculated with L1210 ascites cells. The observations indicate that protocols for delayed treatment of L1210 cells should incorporate a chemotherapeutic phase, similar to induction therapy for acute lymphoblastic leukaemia in man, to reverse the thrombocytopenia and, if possible, the anaemia that is induced by the transplantable tumour. Although our data relate anaemia to the number of tumour cells calculated to be present, chemotherapy did not restore a normal PCV. The reason for the failure to recover a more normal PCV is unknown, though a similar PCV pattern was seen during spontaneous recovery of the platelet count after inoculation of L5178Y cells (Hacker *et al.*, 1977). Our preliminary studies with these doses of VM-26 did not alter the haematocrit of tumour-bearing animals. It is possible that there was insufficient time between treatment and subsequent progression of the disease to allow recovery of the PCV by erythropoiesis.

The drug-induced changes in the leucocyte count provide a simple method for monitoring the response of leukaemia to an anticancer drug. In the present studies, all doses of VM-26 markedly reduced the population of ascites tumour cells in mice given L1210 cells i.p. On the other hand, the peripheral-blood leucocyte count of mice inoculated i.p. with L1210 and treated with VM-26 subsequently increased into the range observed after i.v. inoculation. It is too early to know whether the leucocyte count will predict generally for relapse by systemically located cells. However, the present data do suggest that with VM-26 treatment conversion of L1210 inoculated i.p. into the systemic form associated with i.v. inoculation occurred. This conversion of disease states could lead to a 10-fold error in estimation of the fraction of cells killed by an oncolytic agent. This estimate is based on the fact that i.v. inoculation reduced life-span by about 2 days in comparison with an equivalent number of tumour cells inoculated i.p., a period of time that is about equivalent to a 10-fold variation in the number of cells inoculated i.p. (Skipper *et al.*, 1965).

This work was supported by Research Grant CA 12732, Clinical Center Grant CA 08480 and Training Grant CA 05176 from NCI, National Institutes of Health, Department of Health, Education and Welfare, and by ALSAC.

The authors gratefully acknowledge the gift of VM-26 from Sandoz Pharmaceuticals Division of Sandoz-Warner, Inc.

### REFERENCES

AUSTIN, T., MA, D., DATTA, M. C., ORTEGA, J. A., SHORE, N. A. & DUKES, P. P. (1979) Suppression of mouse erythroid colony formation by L1210 leukemia cells. *Exp. Hematol.*, **7**, 63.

BRECHER, G. & CRONKITE, E. P. (1950). Morphology and enumeration of human blood platelets. *J. Appl. Physiol.*, **3**, 365.

HACKER, M., ROBERTS, D. & JACKSON, C. (1977) Depression of the platelet count after inoculation of mice with L1210 or L5178Y cells. *Br. J. Haematol.*, **35**, 465.

HARRISS, E. B. & HOELZER, D. (1974) Studies of the anaemia in an acute rat leukemia. *Br. J. Haematol.*, **26**, 593.

JACKSON, C. W., KRANCE, R. A., EDWARDS, C. C., WHIDDEN, M. A. & GAUTHIER, P. A. (1980) Shortened platelet survival as a cause of thrombocytopenia in mice with L1210 leukemia. *Cancer Res.*, **40**, 667.

KREMER, W. B. & LASZLO, J. (1973) Hematologic effects of cancer. In *Cancer Medicine*, Eds Holland & Frei. Philadelphia: Lea & Febiger. p. 1085.

LASZLO, J. & KREMER, W. B. (1973) Hematologic effects of chemotherapeutic drugs and radiation. In *Cancer Medicine*, Eds Holland & Frei. Philadelphia: Lea & Febiger. p. 1099.

LAW, L. W., DUNN, T. B., BOYLE, P. J. & MILLER, J. H. (1949) Observations on the effect of a folic acid antagonist on transplantable lymphoid leukemias in mice. *J. Natl Cancer Inst.*, **10**, 179.

SKIPPER, H. E., SCHABEL, F. M., JR, WILCOX, W. S., LASTER, W. R., JR, TRADER, M. W. & THOMPSON, S. A. (1965) Experimental evaluation of potential anticancer agents XVIII. Effects of therapy on viability and rate of proliferation of leukemic cells in various anatomic sites. *Cancer Chemo. Rep.*, **47**, 41.

WINTROBE, M. M., LEE, G. R., BOGGS, D. R., BITHELL, T. C., ATHENS, J. W. & FORESTER, J. (1974) *Clinical Hematology*. Philadelphia: Lea & Febiger.